# Intraoperative changes in whole-blood viscosity in patients undergoing robot-assisted laparoscopic prostatectomy in the steep Trendelenburg position with pneumoperitoneum: a prospective nonrandomized observational cohort study

**DOI:** 10.1186/s12871-019-0919-z

**Published:** 2020-01-07

**Authors:** Jung-Woo Shim, Hyun Kyung Moon, Yong Hyun Park, Misun Park, Jaesik Park, Hyung Mook Lee, Yong-Suk Kim, Young Eun Moon, Sang Hyun Hong, Min Suk Chae

**Affiliations:** 1 Department of Anesthesiology and Pain Medicine, Seoul St. Mary’s Hospital, College of Medicine, The Catholic University of Korea, 222, Banpo-daero, Seocho-gu, Seoul, 06591 Republic of Korea; 2 Department of Urology, Seoul St. Mary’s Hospital, College of Medicine, The Catholic University of Korea, Seoul, Republic of Korea; 3 Department of Biostatistics, Clinical Research Coordinating Center, Catholic Medical Center, The Catholic University of Korea, Seoul, Republic of Korea

**Keywords:** Blood viscosity, Head-down tilt, Pneumoperitoneum, artificial

## Abstract

**Background:**

The aim of this study was to investigate the effect of the steep Trendelenburg position (STP) with pneumoperitoneum on whole-blood viscosity (WBV) in patients undergoing robot-assisted laparoscopic prostatectomy (RALP). The study also analyzed the associations of clinical patient-specific and time-dependent variables with WBV and recorded postoperative outcomes.

**Methods:**

Fifty-eight adult male patients (ASA physical status of I or II) undergoing elective RALP were prospectively analyzed in this study. WBV was intraoperatively measured three times: at the beginning of surgery in the supine position without pneumoperitoneum; after 30 min in the STP with pneumoperitoneum; and at the end of surgery in the supine position without pneumoperitoneum. The WBV at a high shear rate (300 s^− 1^) was recorded as systolic blood viscosity (SBV) and that at a low shear rate (5 s^− 1^) was recorded as diastolic blood viscosity (DBV). Systolic blood hyperviscosity was defined as > 13.0 cP at 300 s^− 1^ and diastolic blood hyperviscosity was defined as > 4.1 cP at 5 s^− 1^.

**Results:**

The WBV and incidences of systolic and diastolic blood hyperviscosity significantly increased from the supine position without pneumoperitoneum to the STP with pneumoperitoneum. When RALP was performed in the STP with pneumoperitoneum, 12 patients (27.3%) who had normal SBV at the beginning of surgery and 11 patients (26.8%) who had normal DBV at the beginning of surgery developed new systolic and diastolic blood hyperviscosity, respectively. The degree of increase in WBV after positioning with the STP and pneumoperitoneum was higher in the patients with hyperviscosity than in those without hyperviscosity at the beginning of surgery. Higher preoperative body mass index (BMI) and hematocrit level were associated with the development of both systolic and diastolic blood hyperviscosity in the STP with pneumoperitoneum. All patients were postoperatively discharged without fatal complications.

**Conclusions:**

Changes in surgical position may influence WBV, and higher preoperative BMI and hematocrit level are independent factors associated with the risk of hyperviscosity during RALP in the STP with pneumoperitoneum.

**Trial registration:**

Clinical Research Information Service, Republic of Korea, approval number: KCT0003295 on October 25, 2018.

## Background

Prostate cancer is one of the most common cancers in the male population, and its incidence gradually increases with age [1]. Based on the characteristics of this cancer, surgical resection is an appropriate option to improve patient outcome. In particular, robot-assisted laparoscopic prostatectomy (RALP) is a technically advanced surgical method that has been widely accepted as feasible and effective due to its various benefits, including its minimally invasive nature, improved prognosis, and favorable functional results [2, 3]. Additionally, RALP has advantages compared to conventional prostatectomy, including higher quality stereoscopic surgical view and increased maneuverability [4]. However, patients who undergo RALP are typically placed in a specific surgical position—the steep Trendelenburg position (STP)—with pneumoperitoneum using CO_2_ gas. This surgical position may be associated with the development of complications, such as subcutaneous emphysema, pulmonary atelectasis, and increased airway and/or optic pressure [5]. Eventually, intraoperative development of these pathophysiological events may become challenging to both urologists and anesthesiologists during RALP.

Whole-blood viscosity (WBV) plays an important role in circulatory flow in both large and small vessels [6]. Because whole blood exhibits non-Newtonian shear-thinning viscosity behavior, such that the velocity of circulatory flow is inversely related to the degree of WBV, the WBV is often regarded as a functional marker of shear stress within vessels [7]. This viscosity is also associated with features of blood components, such as deformability, aggregation, and concentration of red blood cells and other proteins [6, 8, 9]. Because increased WBV may cause greater oscillation of shear stress in relation to endothelial injury, close association of WBV with the development of cardio- and/or cerebrovascular diseases has been reported in clinical settings [10–14]. Additionally, various risk factors, such as obesity, aging, and comorbidity, seem to contribute to increases in WBV [6, 9, 15–18]. Because the age of patients undergoing RALP progressively increases with the prevalence of various comorbidities, such as diabetes mellitus (DM) and hypertension [19], these patients may be vulnerable to the development of hyperviscosity during surgery.

The aim of this study was to investigate the effect of the STP with pneumoperitoneum on WBV in patients undergoing RALP. The study also analyzed the associations of clinical variables with WBV and recorded postoperative outcomes.

## Methods

### Ethical considerations

This single-center, prospective, nonrandomized observational cohort study was approved by the Institutional Review Board of Seoul St. Mary’s Hospital Ethics Committee (approval number: KC18OESI0540). The study protocol was retrospectively/prospectively registered at a publicly accessible clinical registration site that is recognized by the International Committee of Medical Journals Editors (Clinical Research Information Service, Republic of Korea, approval number: KCT0003295, https://cris.nih.go.kr/cris/en/search/search_result_st01.jsp?seq=13745). Written informed consent was obtained from all patients at our hospital who were enrolled between September 2018 and February 2019.

### Study population

Inclusion criteria for this study were male gender, age ≥ 19 years, scheduled for elective RALP, and an American Society of Anesthesiologists (ASA) physical status of I or II [20]. Exclusion criteria were emergency case, age < 19 years, ASA physical status of III–V [20], intraoperative development of hemodynamic instability that required rescue management, such as colloid infusion, blood product transfusion, or strong vasopressor administration (i.e., epinephrine or norepinephrine), and refusal to participate in the study, because patients with symptomatic or uncontrolled diseases may have various confounders related to changing WBV (i.e., possibly, medications and/or nature of own disease). A total of 60 patients agreed to participate in our study; however, 2 patients experienced massive hemorrhage during surgery that required colloid infusion and blood product transfusion, and were thus excluded from the analysis. Therefore, 58 male patients were enrolled in this study.

### Anesthesia and surgery

Balanced anesthesia was performed by attending expert anesthesiologists. Induction of anesthesia was achieved using 1–2 mg.kg^− 1^ propofol (Fresenius Kabi, Bad Homburg, Germany) and 0.6 mg.kg^− 1^ rocuronium (Merck Sharp & Dohme Corp., Kenilworth, NJ, USA); anesthesia was then maintained using 2.0–6.0% desflurane (Baxter, Deerfield, IL, USA) under medical air in oxygen. Remifentanil (Hanlim Pharm. Co., Ltd., Seoul, Republic of Korea) was continuously infused at a rate of 0.1–0.5 μg.kg^− 1^.min^− 1^, as appropriate. The Bispectral Index™ instrument (Medtronic, Minneapolis, MN, USA) was set between 40 and 50 to ensure appropriate hypnotic depth. Rocuronium was repeatedly infused under train-of-four monitoring (> one twitch). End-tidal CO_2_ was set between 30 and 40 mmHg with adjustment of the ventilator mode. Central venous pressure (CVP) was monitored using a central venous catheter (Arrow, Morrisville, NC, USA) that was inserted by experienced radiologists on the day before surgery. For fluid therapy [21], a baseline isotonic crystalloid was prepared based on the estimated fluid maintenance requirements, in turn based on the patient’s weight and anticipated tissue trauma. Additional fluid boluses were infused according to blood loss; however, the total amount of fluid was restricted to a maximum of 1 L before vesicourethral anastomosis.

Surgery was exclusively performed by expert urologists using a specific robot-assisted laparoscopic method. CO_2_ gas was insufflated into the abdominal cavity (i.e., pneumoperitoneum) in the supine position, and patients were placed in the STP with the maximal angle (45°) of the surgical table (Maquet, Rastatt, Germany); this approach was routinely applied to achieve the optimal surgical view. Intra-abdominal pressure was maintained at 12–15 mmHg during surgery. At the time of peritoneal closure, the surgical position was restored to the supine position with removal of CO_2_ gas.

### Measurement of WBV

WBV was measured during surgery three times via a central venous catheter: at the beginning of surgery (i.e., skin incision) in the supine position without pneumoperitoneum, after 30 min in the STP with pneumoperitoneum, and at the end of surgery (i.e., peritoneal closure) in the supine position without pneumoperitoneum (Table 1). Blood samples (3 mL) were collected into evacuated test tubes (BD Vacutainer, K2 EDTA; Becton, Dickinson and Company, Franklin Lakes, NJ, USA) without venous stasis, and measured using an automated scanning capillary tube viscometer (Hemovister; Ubiosis, Seongnam, Republic of Korea): WBV at a high shear rate (300 s^− 1^) was recorded as systolic blood viscosity (SBV) and that at a low shear rate (5 s^− 1^) was recorded as diastolic blood viscosity (DBV) [22]. The reference intervals for WBV were 3.5–4.1 cP at 5 s^− 1^ and 9.4–13.0 cP at 300 s^− 1^ in male patients, based on the manufacturer’s instructions. Accordingly, systolic blood hyperviscosity was defined as > 13.0 cP at 300 s^− 1^ and diastolic blood hyperviscosity was defined as > 4.1 cP at 5 s^− 1^.

**Table 1:** Time points of whole blood viscosity measurements during surgery

Time points of measurements	Whole blood viscosity
1. Awakend patients before anesthetic induction in the supine position	-
2. At beginning of surgery (i.e., skin incision) in the supine position without pneumoperitoneum	O
3. CO_2_ gas insuffluation into abdomen (i.e., pneumoperitoneum) in the supine position	-
4. After 30 min in the steep Trendelenburg position with pneumoperitoneum	O
5. At end of surgery (i.e., peritoneal closure) in the supine position without pneumoperitoneum	O

### Clinical variables

Preoperative factors included age, body mass index (BMI), DM, hypertension, history of current smoking, prostate cancer stage [23], prostate specific antigen, white blood cell (WBC) count, neutrophils, lymphocytes, hematocrit, mean corpuscular volume, mean corpuscular hemoglobin, mean corpuscular hemoglobin concentration, platelet count, glucose, blood urea nitrogen, creatinine, total protein, albumin, sodium, potassium, total cholesterol, triglyceride, high-density -cholesterol (HDL-C), low-density lipoprotein-cholesterol (LDL-C), international normalized ratio, activated partial thrombin time, and fibrinogen. Intraoperative factors included vital signs (i.e., systolic blood pressure [SBP] and diastolic blood pressure [DBP], CVP, body temperature, and heart rate [HR]) at serial surgical positions, total surgical duration, hourly crystalloid infusion, hourly urine output, and total blood loss (measured in a suction bottle). Postoperative findings included total hospital stay and Clavien-Dindo classification at admission [24].

### Statistical analysis

Because of the exploratory nature of this study and the lack of a primary endpoint with anticipated effect sizes, no sample size or statistical power calculations were performed. To achieve robust effects within a reasonable interval of time, we chose to collect data from approximately 60 patients, considering the possibility of patient drop-out. The sample size was based on available data from all patients who underwent elective RALP at our hospital during a 1-year period. A post hoc power analysis revealed that a sample size of 58 was required for an expected power of 99%. The effect size r (*r* = z/sqrt(n)) was 0.82 (|-6.234|/sqrt(58)) and 0.86 (|-6.569|/sqrt(58)), with respect to SBV and DBV. The statistical analysis was calculated using the Wilcoxon signed-rank test (one sample case), as a post hoc method, included in G*Power software 3.1.9.4 (http://www.gpower.hhu.de/). The normality of the distribution of continuous data was evaluated using the Shapiro–Wilk test. Continuous data are expressed as median and interquartile range (IQR), while categorical data are expressed as number and proportion. Serial changes in WBV and vital signs were analyzed using the Friedman’s test and the Wilcoxon signed-rank test with the Bonferroni’s post hoc method. We compared the levels of WBV and vital signs between the beginning of surgery (reference) vs. the STP with pneumoperitoneum and the end of surgery (*p* < 0.025 was statistically significant). After separation of WBV into normal viscosity and hyperviscosity, intraoperative changes in the proportions of patients with hyperviscosity were analyzed using Cochran’s Q test with the McNemar post hoc test. Incidence rates of systolic and diastolic blood hyperviscosity according to surgical position were compared using the *χ*^2^ test or Fisher’s exact test, as appropriate. The associations between clinical variables and the development of whole-blood hyperviscosity during RALP in STP with pneumoperitoneum were evaluated using univariate and multivariate logistic regression analyses. Potentially significant factors (*p* < 0.1) in univariate analyses were entered into multivariate forward and backward regression analyses. The values are expressed as odds ratios and 95% confidence intervals (CIs). The clinically relevant factors were chosen when multiple perioperative factors were inter-correlated. All categorical variables were modeled as dummy variables. The predictive power of independent factors for hyperviscosity were assessed using the area under the receiver operating characteristic curve (AUC). The optimal preoperative BMI and hematocrit cut-off values according to hyperviscosity at the STP with a pneumoperitoneum were determined using the AUC method. All tests were two-sided, and *p* < 0.05 was considered statistically significant. Statistical analyses were performed using SPSS (ver. 22.0 for Windows; SPSS Inc., Chicago, IL, USA) and MedCalc (ver. 11.0 for Windows; MedCalc Software, Mariakerke, Belgium). We used the Strengthening the Reporting of Observational Studies in Epidemiology (STROBE) criteria for our observational study [25].

## Results

### Preoperative and intraoperative findings in patients undergoing RALP

In the present study, all patients had ASA physical status I or II and underwent elective RALP. The median (IQR) age was 69 (62–73) years and the median (IQR) BMI was 25.4 (23.1–26.4) kg.m^− 2^ (Table 2). Eighteen patients (31.0%) had DM and twenty (34.5%) had hypertension; 29 patients (50.0%) were current smokers. With respect to prostate cancer stage, 23 patients (39.7%) were stage I, 28 (48.3%) were stage II, and 7 (12.1%) were stage III. With respect to laboratory variables, the median (IQR) prostate specific antigen was 8.5 (5.3–19.4) ng.mL^− 1^; the median (IQR) WBC count and hematocrit level were 6.2 (5.5–7.4) ×  10^9^.L^− 1^ and 42.9% (40.5–44.8%), respectively, while the median (IQR) platelet count and fibrinogen level were 205.0 (178.5–224.3) × 10^9^.L^− 1^ and 254 (221–290) mg.dL^− 1^, respectively. With respect to lipid profiles, the median (IQR) levels of total cholesterol, triglyceride, HDL-C and LDL-C were 176 (155–198) mg.dL^− 1^, 88 (67–149) mg.dL^− 1^, 50 (42–55) mg.dL^− 1^, and 93 (74–120) mg.dL^− 1^, respectively.

**Table 2:** Preoperative finding in patients who undergoing robot-assisted laparoscopic prostatectomy

Preoperative factor	Whole study cohort
	*n* = 58
Age (years)	69 (62 - 73)
Body mass index (kg.m^-2^)	25.4 (23.1 - 26.4)
Comorbidity	
Diabetes mellitus	18 (31.0%)
Hypertension	20 (34.5%)
Current smoker	29 (50.0%)
Prostate cancer stage	
Stage 1	23 (39.7%)
Stage 2	28 (48.3%)
Stage 3	7 (12.1%)
Laboratory finding	
Prostate specific antigen (ng.mL^-1^)	8.5 (5.3 - 19.4)
WBC count (x 10^9^.L^-1^)	6.2 (5.5 - 7.4)
Neutrophil (%)	57.9 (51.6 - 66.6)
Lymphocyte (%)	29.7 (25.4 - 37.7)
Hematocrit (%)	42.9 (40.5 - 44.8)
MCV (fL)	91.1 (89.3 - 93.4)
MCH (pg)	31.4 (30.5 - 32.4)
MCHC (%)	34.1 (33.3 - 34.8)
Glucose (mg.dL^-1^)	106.0 (94.0 - 113.0)
Blood urea nitrogen (mg.dL^-1^)	15.9 (14.4 - 19.5)
Creatinine (mg.dL^-1^)	0.9 (0.8 - 1.1)
Total Protein (g.dL^-1^)	7.0 (6.7 - 7.3)
Albumin (g.dL^-1^)	4.4 (4.2 - 4.6)
Sodium (mEq.L^-1^)	142 (140 - 144)
Potassium (mEq.L^-1^)	4.5 (4.3 - 4.8)
Platelet count ( x 10^9^.L^-1^)	205.0 (178.5 - 224.3)
International normalized ratio	1.1 (1.0 - 1.1)
Activated PTT (sec)	26.6 (25.2 - 27.6)
Fibrinogen (mg.dL^-1^)	254 (221 - 290)
Total cholesterol (mg.dL^-1^)	176 (155 - 198)
Triglyceride (mg.dL^-1^)	88 (67 - 149)
HDL-Cholesterol (mg.dL^-1^)	50 (42 - 55)
LDL-Cholesterol (mg.dL^-1^)	93 (74 - 120)

Values are expressed as median (interquartile) and number (proportion)

*Abbreviations*: *WBC* white blood cell, *MCV* mean corpuscular volume, *MCH* mean corpuscular hemoglobin, *MCHC* mean corpuscular hemoglobin concentration, *HDL* high-density lipoprotein, *LDL* low-density lipoprotein

The median (IQR) surgical duration was 130 (120–145) min, and the median (IQR) levels of crystalloid infusion, urine output, and blood loss were 2.3 (1.8–3.3) mL.kg^− 1^.h^− 1^, 0.7 (0.6–0.8) mL.kg^− 1^.h^− 1^, and 25 (13–25) mL, respectively (Table 3). With respect to vital signs, SBP, DBP, and CVP significantly increased, while HR decreased, from the supine position without pneumoperitoneum (beginning of surgery) to the STP with pneumoperitoneum. There was no difference in vital signs between the beginning and end of surgery when both were assessed in the supine position without pneumoperitoneum.

**Table 3:** Intraoperative finding in patients who undergoing robot-assisted laparoscopic prostatectomy

Intraoperative factor	Whole study cohort
	n = 58
Surgical duration (min)	130 (120 - 145)
Crystalloid infusion (mL.kg^-1^.h^-1^)	2.3 (1.8 - 3.3)
Urine output (mL.kg^-1^.h^-1^)	0.7 (0.6 - 0.8)
Bleeding loss (mL)	25 (13 - 25)
Vital signs	
Supine position without pneumoperitoneum (beginning of surgery)	
Systolic blood pressure (mmHg)	102 (92 - 115)
Diastolic blood pressure (mmHg)	67 (61 - 72)
Central venous pressure (mmHg)	4 (3 - 6)
Body temperature (°C)	36.3 (36.2 - 36.5)
Heart rate (beats.min^-1^)	66 (57 - 72)
Steep Trendelenburg position with pneumoperitoneum	
Systolic blood pressure (mmHg)	113 (103 - 125)^**^
Diastolic blood pressure (mmHg)	74 (65 - 81)^**^
Central venous pressure (mmHg)	19 (15 - 21)^***^
Body temperature (°C)	36.3 (36.0 - 36.5)
Heart rate (beats.min^-1^)	61 (53 - 70)^*^
Supine position without pneumoperitoneum (end of surgery)	
Systolic blood pressure (mmHg)	106 (101 - 111)
Diastolic blood pressure (mmHg)	70 (61 - 76)
Central venous pressure (mmHg)	5 (3 - 6)
Body temperature (°C)	36.3 (36.0 - 36.5)
Heart rate (beats.min^-1^)	62 (56 - 72)

Values are expressed as median and interquartile

^*^p<0.025 based on the level at the beginning of surgery

^**^p<0.01 based on the level at the beginning of surgery

^***^p<0.001 based on the level at the beginning of surgery

### Intraoperative changes in SBV and DBV

Levels of SBV and DBV significantly increased from the supine position without pneumoperitoneum (beginning of surgery) to the STP with pneumoperitoneum (Table 4). The incidence rates of systolic and diastolic blood hyperviscosity were higher in the STP with pneumoperitoneum than in the supine position without pneumoperitoneum (beginning of surgery). However, there were no differences in the levels of SBV and DBV and incidence rates of systolic and diastolic blood hyperviscosity between the beginning and end of surgery when both were assessed in the supine position without pneumoperitoneum.

**Table 4:** Changes in systolic and diastolic blood viscosities during robot-assisted laparoscopic prostatectomy

	Systolic blood viscosity	Diastolic blood viscosity
Level (cP)	n=58	n=58
Supine position without pneumoperitoneum (beginning of surgery)	3.9 (3.6 - 4.1)	12.1 (11.0 - 13.3)
Steep Trendelenburg position with pneumoperitoneum	4.1 (3.8 - 4.9)^***^	12.9 (12.0 - 16.4)^***^
Supine position without pneumoperitoneum (end of surgery)	3.8 (3.5 - 4.1)	11.6 (10.5 - 13.0)
Incidence (%)	Systolic blood hyperviscosity	Diastolic blood hyperviscosity
Supine position without pneumoperitoneum (beginning of surgery)	14/58 (24.1%)	17/58 (29.3%)
Steep Trendelenburg position with pneumoperitoneum	26/58 (44.8%)^***^	28/58 (48.3%)^**^
Supine position without pneumoperitoneum (end of surgery)	12/58 (20.7%)	14/58 (24.1%)

Values are expressed as median (interquartile) and number (proportion)

*Abbreviation*: *cP* centipoise

^*^p<0.025 based on the level at the beginning of surgery

^**^p<0.01 based on the level at the beginning of surgery

^***^p<0.001 based on the level at the beginning of surgery

With respect to changes in the prevalence rates of systolic and diastolic blood hyperviscosity during surgery (Table 5), 12 patients (27.3%) who had normal SBV in the supine position without pneumoperitoneum (beginning of surgery) showed new-onset systolic blood hyperviscosity in the STP with pneumoperitoneum. In addition, 11 patients (26.8%) who had normal DBV in the supine position without pneumoperitoneum (beginning of surgery) showed new-onset diastolic blood hyperviscosity in the STP with pneumoperitoneum. All patients with systolic (*n* = 14) or diastolic (*n* = 17) blood hyperviscosity in the supine position without pneumoperitoneum (beginning of surgery) continued to show hyperviscosity in the STP with pneumoperitoneum. In patients with persistent hyperviscosity, the changes in SBV (*n* = 14) were as follows: 4.4 (4.2–5.1) cP in the supine position without pneumoperitoneum (beginning of surgery), 5.3 (4.9–5.5) cP in the STP with pneumoperitoneum, and 4.3 (4.1–5.3) cP in the supine position without pneumoperitoneum (end of surgery). Furthermore, in patients with persistent hyperviscosity, the changes in DBV (*n* = 17) were as follows: 13.5 (13.4–16.5) cP in the supine position without pneumoperitoneum (beginning of surgery), 17.1 (16.4–17.6) cP in the STP with pneumoperitoneum, and 13.5 (12.9–16.3) cP in the supine position without pneumoperitoneum (end of surgery). There were significant differences in SBV (*p* = 0.001) and DBV (*p* < 0.001) between the supine position without pneumoperitoneum (beginning of surgery) and the STP with pneumoperitoneum.

**Table 5:** Changes in incidences of systolic and diastolic blood hyperviscosities during robot-assisted laparoscopic prostatectomy

Systolic blood viscosity			
	In the supine position without pneumoperitoneum (beginning of surgery)		
	Normal viscosity (n = 44)	Hyperviscosity (n = 14)	*p*
In the steep Trendelenburg position with pneumoperitoneum			<0.001
Normal viscosity	32/44 (72.7%)	0/14 (0.0%)	
Hyperviscority	12/44 (27.3%)	14/14 (100.0%)	
In the supine position without pneumoperitoneum (end of surgery)			<0.001
Normal viscosity	42/44 (95.5%)	4/14 (28.6%)	
Hyperviscority	2/44 (4.5%)	10/14 (71.4%)	
Diastolic blood viscosity			
	In the supine position without pneumoperitoneum (beginning of surgery)		
	Normal viscosity (n = 41)	Hyperviscosity (n = 17)	*p*
In the steep Trendelenburg position with pneumoperitoneum			<0.001
Normal viscosity	30/41 (73.2%)	0/17 (0.0%)	
Hyperviscosity	11/41 (26.8%)	17/17 (100.0%)	
In the supine position without pneumoperitoneum (end of surgery)			<0.001
Normal viscosity	39/41 (95.1%)	5/17 (29.4%)	
Hyperviscosity	2/41 (4.9%)	12/17 (70.6%)	

WBV significantly increased from the supine position without pneumoperitoneum to the STP with pneumoperitoneum in patients with/without hyperviscosity at the beginning of surgery (i.e., supine position without pneumoperitoneum) (Additional files 1 and 2); however, the degree of increase in WBV after positioning with the STP and pneumoperitoneum was higher in patients with hyperviscosity than in those without hyperviscosity.

### Association of the development of systolic and diastolic blood hyperviscosity with clinical factors in patients undergoing RALP in the STP with pneumoperitoneum

With respect to systolic blood hyperviscosity in the STP with pneumoperitoneum (Table 6), preoperative findings (i.e., age, BMI, hypertension, hematocrit level, and blood urea nitrogen level) and one intraoperative finding (i.e., CVP in the STP with pneumoperitoneum) were potentially associated with the development of hyperviscosity in univariate analysis. Higher preoperative BMI and hematocrit level were significantly associated with the development of hyperviscosity in multivariate analysis. The predictive accuracy of this model was good (AUC = 0.821; 95% CI = 0.708–0.934; *p* < 0.001). The optimal cut-off values for preoperative BMI and hematocrit were 25.8 kg.m^− 2^ (AUC = 0.649; 95% CI = 0.513–0.770; *p* = 0.042) and 43% (AUC = 0.754; 95% CI = 0.623–0.858; *p* < 0.001), respectively, for hyperviscosity.

**Table 6:** Association of development in systolic blood hyperviscosity with clinical factors in patients in the steep Trendelenburg position with pneumoperitoneum during surgery

	Univariate logistic regression				Multivariate logistic regression			
	*ß*	Odds ratio	95% CI	*p*	*ß*	Odds ratio	95% CI	*p*
Preoperative finding								
Age (years)	-0.079	0.924	0.851 - 1.003	0.060				
Body mass index (kg.m^-2^)	0.218	1.244	0.994 - 1.555	0.056	0.417	1.517	1.114 - 2.067	0.008
Comorbidity								
Diabetes mellitus	-0.693	0.500	0.157 - 1.594	0.241				
Hypertension	0.968	2.633	0.915 - 7.576	0.073				
Current smoker	0.561	1.753	0.616 - 4.988	0.293				
Prostate cancer stage								
Stage 1	reference			0.069				
Stage 2	1.262	3.532	1.099 - 11.358	0.034				
Stage 3	-0.090	0.914	0.142 - 5.902	0.925				
Laboratory finding								
Prostate specific antigen (ng.mL^-1^)	0.015	1.015	0.990 - 1.040	0.237				
WBC count (x 10^9^.L^-1^)	0.100	1.106	0.810 - 1.509	0.527				
Neutrophil (%)	0.011	1.011	0.944 - 1.084	0.748				
Lymphocyte (%)	-0.034	0.966	0.893 - 1.046	0.394				
Hematocrit (%)	0.388	1.474	1.147 - 1.895	0.002	0.521	1.684	1.218 - 2.327	0.002
MCV (fL)	-0.019	0.981	0.831 - 1.159	0.826				
MCH (pg)	0.060	1.062	0.702 - 1.605	0.776				
MCHC (%)	0.507	1.660	0.862 - 3.196	0.129				
Platelet count ( x 10^9^.L^-1^)	0.009	1.009	0.997 - 1.021	0.160				
Glucose (mg.dL^-1^)	0.005	1.005	0.986 - 1.024	0.617				
Blood urea nitrogen (mg.dL^-1^)	-0.128	0.880	0.776 - 0.998	0.047				
Creatinine (mg.dL^-1^)	-2.422	0.089	0.005 - 1.703	0.108				
Total Protein (g.dL^-1^)	0.960	2.612	0.619 - 11.024	0.191				
Albumin (g.dL^-1^)	1.496	4.465	0.622 - 32.052	0.137				
Sodium (mEq.L^-1^)	-0.066	0.936	0.735 - 1.191	0.589				
Potassium (mEq.L^-1^)	0.255	1.291	0.243 - 6.866	0.765				
Total cholesterol (mg.dL^-1^)	0.003	1.003	0.981 - 1.024	0.809				
Triglyceride (mg.dL^-1^)	0.000	1.000	0.99 - 1.009	0.956				
HDL-Cholesterol (mg.dL^-1^)	-0.025	0.975	0.896 - 1.061	0.562				
LDL-Cholesterol (mg.dL^-1^)	-0.001	0.999	0.973 - 1.026	0.949				
International normalized ratio	-0.608	0.545	0.001 - 291.772	0.850				
Activated PTT (sec)	0.061	1.063	0.867 - 1.304	0.556				
Fibrinogen (mg.dL^-1^)	0.004	1.004	0.993 - 1.014	0.502				
Intraoperative finding								
Surgical duration (min)	-0.007	0.993	0.970 - 1.017	0.575				
Vital signs in the supine position without pneumoperitoneum (beginning of surgery)								
Systolic blood pressure (mmHg)	0.007	1.007	0.977 - 1.037	0.659				
Diastolic blood pressure (mmHg)	0.042	1.043	0.992 - 1.096	0.102				
Central venous pressure (mmHg)	0.184	1.202	0.933 - 1.548	0.155				
Body temperature (°C)	-0.887	0.412	0.053 - 3.200	0.396				
Heart rate (beats.min^-1^)	0.041	1.042	0.989 - 1.099	0.124				
Vital signs in the steep Trendelenburg position with pneumoperitoneum								
Systolic blood pressure (mmHg)	0.014	1.014	0.980 - 1.050	0.417				
Diastolic blood pressure (mmHg)	0.042	1.043	0.991 - 1.098	0.107				
Central venous pressure (mmHg)	0.115	1.122	0.983 - 1.280	0.089				
Body temperature (°C)	0.363	1.438	0.293 - 7.051	0.654				
Heart rate (beats.min^-1^)	0.000	1.000	0.951 - 1.052	0.999				
Crystalloid infusion (mL.kg^-1^.h^-1^)	-0.238	0.788	0.528 - 1.178	0.246				
Urine output (mL.kg^-1^.h^-1^)	-1.316	0.268	0.030 - 2.378	0.237				
Bleeding loss (mL)	-0.003	0.997	0.988 - 1.005	0.459				

*Abbreviations*: *WBC* white blood cell, *MCV* mean corpuscular volume, *MCH* mean corpuscular hemoglobin, *MCHC* mean corpuscular hemoglobin concentration, *HDL* high-density lipoprotein, *LDL* low-density lipoprotein

With respect to diastolic blood hyperviscosity in the STP with pneumoperitoneum (Table 7), preoperative findings (i.e., age, BMI, and hematocrit level) and intraoperative findings (i.e., DBP and HR in the supine position without pneumoperitoneum [beginning of surgery]) were potentially associated with the development of hyperviscosity in univariate analysis. Higher preoperative BMI and hematocrit level were significantly associated with the development of hyperviscosity in multivariate analysis. The predictive accuracy of this model was fair (AUC = 0.790; 95% CI = 0.673–0.908; *p* < 0.001). The optimal cut-off values for preoperative BMI and hematocrit were 25.1 kg.m^− 2^ (AUC = 0.645; 95% CI = 0.509–0.766; *p* = 0.046) and 43% (AUC = 0.712; 95% CI = 0.579–0.824; *p* = 0.002), respectively, for hyperviscosity.

**Table 7:** Association of development in diastolic blood hyperviscosity with clinical factors in patients in the steep Trendelenburg position with pneumoperitoneum during surgery

	Univariate logistic regression				Multivariate logistic regression			
	*ß*	Odds ratio	95% CI	*p*	*ß*	Odds ratio	95% CI	*p*
Preoperative finding								
Age (years)	-0.081	0.922	0.849 - 1.000	0.051				
Body mass index (kg.m^-2^)	0.221	1.247	0.998 - 1.558	0.052	0.269	1.309	1.021 - 1.678	0.034
*Comorbidity*								
Diabetes mellitus	-0.894	0.409	0.128 - 1.307	0.131				
Hypertension	0.764	2.147	0.758 - 6.087	0.150				
Current smoker	0.556	1.744	0.616 - 4.933	0.295				
Prostate cancer stage								
Stage 1	reference			0.181				
Stage 2	1.064	2.898	0.922 - 9.108	0.069				
Stage 3	0.341	1.406	0.250 - 7.896	0.699				
Laboratory finding								
Prostate specific antigen (ng.mL^-1^)	0.016	1.016	0.990 - 1.043	0.222				
WBC count (x 10^9^.L^-1^)	0.060	1.061	0.780 - 1.444	0.705				
Neutrophil (%)	0.011	1.011	0.944 - 1.084	0.748				
Lymphocyte (%)	-0.034	0.966	0.893 - 1.046	0.394				
Hematocrit (%)	0.317	1.373	1.093 - 1.725	0.007	0.333	1.395	1.095 - 1.777	0.007
MCV (fL)	-0.019	0.981	0.831 - 1.159	0.826				
MCH (pg)	0.060	1.062	0.702 - 1.605	0.776				
MCHC (%)	0.507	1.660	0.862 - 3.196	0.129				
Platelet count ( x 10^9^.L^-1^)	0.005	1.005	0.994 - 1.017	0.396				
Glucose (mg.dL^-1^)	0.002	1.002	0.984 - 1.021	0.813				
Blood urea nitrogen (mg.dL^-1^)	-0.089	0.915	0.820 - 1.021	0.111				
Creatinine (mg.dL^-1^)	-1.683	0.186	0.014 - 2.388	0.196				
Total Protein (g.dL^-1^)	0.516	1.675	0.420 - 6.688	0.465				
Albumin (g.dL^-1^)	1.249	3.485	0.506 - 23.998	0.205				
Sodium (mEq.L^-1^)	-0.123	0.884	0.692 - 1.130	0.325				
Potassium (mEq.L^-1^)	0.156	1.169	0.222 - 6.154	0.854				
Total cholesterol (mg.dL^-1^)	0.005	1.005	0.983 - 1.026	0.678				
Triglyceride (mg.dL^-1^)	0.008	1.008	0.995 - 1.020	0.224				
HDL-Cholesterol (mg.dL^-1^)	-0.028	0.972	0.894 - 1.057	0.508				
LDL-Cholesterol (mg.dL^-1^)	0.000	1.000	0.975 - 1.026	0.995				
International normalized ratio	-1.317	0.268	0.000 - 153.896	0.685				
Activated PTT (sec)	0.045	1.046	0.910 - 1.201	0.529				
Fibrinogen (mg.dL^-1^)	0.002	1.002	0.991 - 1.012	0.747				
Intraoperative finding								
Surgical duration (min)	-0.008	0.992	0.969 - 1.015	0.505				
Vital signs in the supine position without pneumoperitoneum (beginning of surgery)								
Systolic blood pressure (mmHg)	0.015	1.015	0.985 - 1.047	0.324				
Diastolic blood pressure (mmHg)	0.054	1.055	1.001 - 1.112	0.045				
Central venous pressure (mmHg)	0.203	1.225	0.949 - 1.582	0.119				
Body temperature (°C)	-1.183	0.306	0.039 - 2.417	0.262				
Heart rate (beats.min^-1^)	0.052	1.053	0.998 - 1.112	0.060				
Vital signs in the steep Trendelenburg position with pneumoperitoneum								
Systolic blood pressure (mmHg)	0.005	1.005	0.971 - 1.040	0.778				
Diastolic blood pressure (mmHg)	0.039	1.040	0.988 - 1.094	0.131				
Central venous pressure (mmHg)	0.095	1.099	0.966 - 1.251	0.149				
Body temperature (°C)	0.098	1.103	0.228 - 5.339	0.903				
Heart rate (beats.min^-1^)	0.017	1.017	0.966 - 1.070	0.516				
Crystalloid infusion (mL.kg^-1^.h^-1^)	-0.229	0.795	0.537 - 1.178	0.254				
Urine output (mL.kg^-1^.h^-1^)	-1.467	0.231	0.026 - 2.054	0.189				
Bleeding loss (mL)	0.001	1.001	0.992 - 1.009	0.896				

*Abbreviations*: *WBC* white blood cell, *MCV* mean corpuscular volume, *MCH* mean corpuscular hemoglobin, *MCHC* mean corpuscular hemoglobin concentration, *HDL* high-density lipoprotein, *LDL* low-density lipoprotein

### Observed complications

Nearly all patients in our study were discharged on postoperative day 6 (mean ± standard deviation: 6.2 ± 0.8 days) and there were no fatal complications representative of Clavien-Dindo class I [24].

## Discussion

The main finding of this study was that performance of RALP in the STP with pneumoperitoneum may contribute to a significant increase in WBV (in either SBV or DBV), which subsequently results in the development of hyperviscosity. Additionally, the surgical position change with gas insufflation may have aggravated WBV more in patients with hyperviscosity at the beginning of surgery than in those without hyperviscosity. Higher preoperative BMI and hematocrit level were significantly associated with increases in both SBV and DBV when RALP was performed in the STP with pneumoperitoneum.

WBV has been linked to complicated interactions among blood components, such as red blood cells, fibrinogen, and lipid profiles. Moreover, WBV is separated into SBV and DBV, based on shear rates: SBV exhibits a high shear rate (≥ 300 s^− 1^) and DBV exhibits a low shear rate (≤ 5 s^− 1^) [14, 26, 27]. SBV is related to the frictional characteristics of rapid blood flow in larger vessels, which are affected by inertia force, rather than viscous force [14, 26]. Notably, hematocrit and erythrocyte deformability mainly contribute to increased SBV [28]. In contrast, DBV is related to the frictional characteristics of low blood flow in smaller vessels (i.e., distal or peripheral vascular segments), which are affected by viscous force, rather than inertia force; notably, DBV predominantly determines tissue perfusion [14]. Plasma proteins and lipids, such as fibrinogen and LDL-C, largely contribute to increased DBV, together with hematocrit and red blood cell aggregation [29].

Many clinical studies have suggested that elevated WBV is associated with the development of cardiovascular and cerebrovascular diseases [10, 11, 13, 30]. In patients with mitral annular calcification, higher WBV was predictive of pathologic mitral annulus. Notably, viscosity was inversely correlated with early and late diastolic mitral annular velocity, which are represented by mitral annular limitations [13]. In patients with acute ischemic stroke, increased DBV was significantly associated with the prevalence of stroke after adjustment for various vascular risk factors, such as age and sex; viscosity was also correlated with hematocrit level at admission. Notably, viscosity decreased after hydration therapy, but hyperviscosity recurred when hydration therapy was discontinued [11]. Our study population showed a more stable clinical condition before surgery, as represented by ASA physical status I or II, compared to the patients in previous studies [10, 11, 13, 20, 30]. During RALP surgery, we encountered no hemodynamic instability that required aggressive fluid or blood product transfusion therapy. However, the change in surgical condition (i.e., to STP with pneumoperitoneum) led to dramatic increases in both intra-abdominal/thoracic pressure (i.e., CVP) and WBV (i.e., SBV and DBV); and caused more stickiness of WBV in the patients with hyperviscosity at the beginning of surgery than in those without hyperviscosity. Therefore, establishing intraoperative STP with a pneumoperitoneum may contribute to changes in WBV. Subsequently, when patients were returned to the supine position without pneumoperitoneum, both intra-abdominal/thoracic pressure and WBV were reduced. Although there was a lack of clinical endpoints (i.e., morbidity and/or mortality) in our study, our results demonstrate a relationship between intraoperative WBV and surgical STP position with gas insufflation, which is usually present during laparoscopy-based surgery [5], and the impact of surgical position with gas insufflation on aggravation of the WBV condition in a relatively healthy population (i.e., ASA physical status of I or II). Although further study is required, these findings imply that patients with hyperviscosity are more susceptible to aggravated WBV than those without hyperviscosity when the STP with pneumoperitoneum is performed during surgery.

The mechanism by which the WBV is increased in the STP with pneumoperitoneum remains unclear. In the present study, higher BMI and hematocrit level before surgery were significantly related with increases in both SBV and DBV in the STP with pneumoperitoneum. First, obesity has been linked to increases in hemorheological parameters, including WBV [18, 31]. In obese patients without comorbidities, significant increases in blood viscosity have been observed, which may be related to poor erythrocyte aggregation and deformability [9]. In a population of female volunteers, blood viscosity was positively correlated with overall body fat status, as measured by BMI; in addition, increased BMI was correlated with increased red blood cell rigidity [32]. Our study findings were consistent with those of previous studies [9, 18, 31, 32], in that elevated BMI was significantly associated with increased WBV. However, our findings differed from those of previous studies [9, 18, 31, 32]: WBV in previous study populations was evaluated in the resting supine position, whereas in our study, WBV was serially evaluated according to changes in surgical position with/without pneumoperitoneum, which may affect systemic circulatory pressure or flow [33–35]. Therefore, increased WBV was more pronounced in the STP with pneumoperitoneum than in the supine position without pneumoperitoneum; this finding may be due to increasing intra-abdominal/thoracic pressure, which led to the compression of vessels related to impairment of circulatory flow [17, 33, 36, 37]. Compared to non-obese patients, obese patients in the STP with pneumoperitoneum showed increased pressure of abdominal contents on cardiovascular circulatory flow more frequently [38]. Although further studies are needed to identify the potential cascade underlying the development of hyperviscosity, higher BMI may have a greater impact on increased blood viscosity during RALP in the STP with pneumoperitoneum.

Hematocrit is an important factor that is positively related to WBV [8]. In the clinical setting, increases in WBV and hematocrit level have been associated with the risk of cardiovascular disease. In particular, small vessel flow and perfusion, such as coronary collateral circulation, is most affected by elevated WBV [12]. Because high WBV through wall shear stress seems to be directly related to the rupture or erosion of weak plaques, therapeutic phlebotomy to decrease hematocrit may select for patients at risk of atherosclerotic occlusions. Additionally, monitoring of WBV was useful for selection of appropriate time points to perform phlebotomy, which could reduce hematocrit level and, subsequently, WBV [39]. However, previously, the associations among hematocrit, WBV, and surgical position were not investigated. In the operating theater, patients are required to assume various positions that are not physiologically normal, such as the STP. Furthermore, consistent with advanced surgical methods, including robot-assisted laparoscopic surgery, CO_2_ gas insufflation in the abdominal cavity (i.e., pneumoperitoneum) is used to achieve an optimal surgical view, but this may impair hemodynamic flow [33, 35, 36]. In the present study, the relationship between hematocrit and WBV may have been strengthened in the STP with pneumoperitoneum, compared to the supine position without pneumoperitoneum. This hypothesis was supported by the observation that more than one-quarter of patients with normal viscosity showed development of hyperviscosity after changing position, from the supine position without pneumoperitoneum to the STP with pneumoperitoneum. At the end of surgery (i.e., the supine position without pneumoperitoneum), the WBV recovered to the level observed at the beginning of surgery (i.e., the supine position without pneumoperitoneum). Additionally, in patients who showed hyperviscosity at the beginning of surgery (i.e., the supine position without pneumoperitoneum), the WBV further increased in the STP with pneumoperitoneum, and then decreased at the end of surgery (i.e., the supine position without pneumoperitoneum).

There were some limitations to this study. First, because of logistical restrictions, we did not evaluate the WBV prior to surgery. Neither hemodynamic instability nor resuscitation therapy were observed before or after anesthetic induction in our cohort. Second, we did not measure the deformability of red blood cells. Because red blood cell deformability affects WBV [28, 29], further analyses may be helpful to identify potential causes of increased WBV during surgery in the STP with pneumoperitoneum, and to subsequently restore normal viscosity in the supine position without pneumoperitoneum. Third, we excluded patients who had uncontrolled comorbidities (ASA physical status III or IV), such as DM and hypertension [20]. All patients were discharged without fatal complications (i.e., Clavien-Dindo class I) [24]. Our exclusion criteria may have precluded an effect of comorbidities on WBV and postoperative outcomes [40]. Therefore, an additional large-scale cohort study of patients undergoing RALP is needed to evaluate the association between STP and WBV, and to make suggestions with respect to the management of increased WBV during surgery.

## Conclusions

We found that WBV (i.e., SBV and DBV) significantly increased, in a time-dependent manner, in patients undergoing RALP in the STP with pneumoperitoneum. The WBV reached levels indicative of hyperviscosity, but no patient showed occlusions of hemodynamic circulation, which can result from the recovery of normal WBV at the end of surgery (i.e., the supine position without pneumoperitoneum). The patients with hyperviscosity at the beginning of surgery (i.e., the supine position without pneumoperitoneum) seemed to be more susceptible to aggravation of WBV than those without hyperviscosity when the STP with pneumoperitoneum was performed during surgery. Therefore, changes in surgical position may contribute to changes in WBV. Higher preoperative BMI and hematocrit level were significantly associated with the development of hyperviscosity during RALP in the STP with pneumoperitoneum. An additional large-scale study may be helpful for identification of patients at risk of hyperviscosity and circulatory impairment during surgery requiring use of the STP with pneumoperitoneum.

## Supplementary information

**Additional file 1.** Intraoperative systolic blood viscosity between patients with/without hyperviscosity at the beginning of surgery.

**Additional file 2.** Intraoperative diastolic blood viscosity between patients with/without hyperviscosity at the beginning of surgery.

## Data Availability

The datasets used and/or analyzed during this study are available from the corresponding author on reasonable request.

## Abbreviations

ASA: American Society of Anesthesiologists
BMI: Body mass index
CVP: Central venous pressure
DBV: Diastolic blood viscosity
DM: Diabetes mellitus
HDL: High-density lipoprotein
HR: Heart rate
LDL: Low-density lipoprotein
RALP: Robot-assisted laparoscopic prostatectomy
SBV: Systolic blood viscosity
STP: Steep Trendelenburg position
WBC: White blood cell
WBV: Whole blood viscosity
