# Supplementary material for: Intraoperative changes in whole-blood viscosity in patients undergoing robot-assisted laparoscopic prostatectomy in the steep Trendelenburg position with pneumoperitoneum: a prospective nonrandomized observational cohort study
Source: BMC Anesthesiol. 2020 Jan 7;20:7. doi: 10.1186/s12871-019-0919-z (PMC6947909; doi:10.1186/s12871-019-0919-z)
Supplement: Supplementary file 1 — Additional file 1. Intraoperative systolic blood viscosity between patients with/without hyperviscosity at the beginning of surgery. [file 12871_2019_919_MOESM1_ESM.docx]

**Additional file**

| **Additional file 1.** Intraoperative systolic blood viscosity between patients with/without hyperviscosity at the beginning of surgery (DOCX) | | | |
| --- | --- | --- | --- |
| **Group** | **Normal viscosity** | **Hyperviscosity** | ***p*** |
| **n** | **44** | **14** |  |
| **Level of systolic blood viscosity (cP)** | | | |
| *Supine position without pneumoperitoneum*  *(beginning of surgery)* | 3.8 (3.6 – 4.0) | 4.4 (4.2 – 5.1) | <0.001 |
| *Steep Trendelenburg position with*  *pneumoperitoneum* | 4.0 (3.7 – 4.2)^***^ | 5.3 (4.9 – 5.5)^**^ | <0.001 |
| *Supine position without pneumoperitoneum*  *(end of surgery)* | 3.7 (3.5 – 3.9) | 4.3 (4.1 – 5.3) | <0.001 |
| **Change of systolic blood viscosity (%)^‡^** | | | |
| *Supine position without pneumoperitoneum*  *(beginning of surgery)* | **Reference** | **Reference** |  |
| *Steep Trendelenburg position with*  *pneumoperitoneum* | 5.3 (2.6 – 10.9) | 10.6 (6.7 – 19.0) | 0.016 |
| *Supine position without pneumoperitoneum*  *(end of surgery)* | 0.0 (-4.4 – 2.6) | -2.1 (-5.0 – 0.9) | 0.913 |
| **Abbreviation:** cP, centipoise ^*^*p*<0.025 based on the level at the beginning of surgery  ^**^*p*<0.01 based on the level at the beginning of surgery ^***^*p*<0.001 based on the level at the beginning of surgery  ^‡^Change (%) of systolic blood viscosity based on the level at the beginning of surgery  **NOTE:** Values are expressed as median and interquartile. | | | |
