# Supplementary material for: Intraoperative changes in whole-blood viscosity in patients undergoing robot-assisted laparoscopic prostatectomy in the steep Trendelenburg position with pneumoperitoneum: a prospective nonrandomized observational cohort study
Source: BMC Anesthesiol. 2020 Jan 7;20:7. doi: 10.1186/s12871-019-0919-z (PMC6947909; doi:10.1186/s12871-019-0919-z)
Supplement: Supplementary file 2 — Additional file 2. Intraoperative diastolic blood viscosity between patients with/without hyperviscosity at the beginning of surgery. [file 12871_2019_919_MOESM2_ESM.docx]

**Additional file**

| **Additional file 2.** Intraoperative diastolic blood viscosity between patients with/without hyperviscosity at the beginning of surgery (DOCX) | | | |
| --- | --- | --- | --- |
| **Group** | **Normal viscosity** | **Hyperviscosity** | ***p*** |
| **n** | **41** | **17** |  |
| **Level of diastolic blood viscosity (cP)** | | | |
| *Supine position without pneumoperitoneum*  *(beginning of surgery)* | 11.5 (10.5 – 12.3) | 13.5 (13.4 – 16.5) | <0.001 |
| *Steep Trendelenburg position with*  *pneumoperitoneum* | 12.4 (11.7 – 13.1)^***^ | 17.1 (16.4 – 17.6)^***^ | <0.001 |
| *Supine position without pneumoperitoneum*  *(end of surgery)* | 11.1 (10.2 – 11.9) | 13.5 (12.9 – 16.3) | <0.001 |
| **Change of diastolic blood viscosity (%)^‡^** | | | |
| *Supine position without pneumoperitoneum*  *(beginning of surgery)* | **Reference** | **Reference** |  |
| *Steep Trendelenburg position with*  *pneumoperitoneum* | 6.9 (4.3 – 13.5) | 18.2 (8.5 – 27.3) | 0.013 |
| *Supine position without pneumoperitoneum*  *(end of surgery)* | -0.9 (-6.8 – 3.1) | -0.7 (-7.7 – 2.3) | 0.620 |
| **Abbreviation:** cP, centipoise ^*^*p*<0.025 based on the level at the beginning of surgery  ^**^*p*<0.01 based on the level at the beginning of surgery ^***^*p*<0.001 based on the level at the beginning of surgery  ^‡^Change (%) of diastolic blood viscosity based on the level at the beginning of surgery  **NOTE:** Values are expressed as median and interquartile. | | | |
